# Case report: Secretory carcinoma of the breast with ETV6-NTRK3 fusion: clinicopathological features and molecular confirmation by FISH in two cases

**DOI:** 10.3389/fonc.2026.1883805

**Published:** 2026-07-02

**Authors:** Xiaoyu Sun, Jiaqi Liu

**Affiliations:** 1 Department of Pathology, Zibo Central Hospital, Zibo, Shandong, China; 2 Department of Breast and Thyroid Surgery, Zibo Central Hospital, Zibo, Shandong, China

**Keywords:** breast, case report, ETV6-NTRK3, molecular pathology, secretory carcinoma, triple-negative breast cancer

## Abstract

**Background:**

Secretory carcinoma of the breast (SCB) is an extremely rare malignant neoplasm, accounting for less than 0.15% of all breast carcinomas. It is characterized by distinctive secretory activity, a frequently triple-negative immunophenotype, and recurrent ETV6-NTRK3 fusion (1–3).

**Case presentations:**

We report two Chinese women with SCB. Case 1 involved a 55-year-old woman with a 1.2-cm mass, and Case 2 involved a 43-year-old woman with a 3.8-cm mass. Both tumors showed characteristic morphology, including microcystic/tubulocystic, solid, tubular, and focally papillary growth patterns, abundant eosinophilic secretory material, granular eosinophilic or vacuolated cytoplasm, and minimal cytologic atypia. Immunohistochemistry revealed a triple-negative phenotype (ER-negative, PR-negative, and HER2-negative) with diffuse S-100 and CK5/6 positivity. Mammaglobin and MUC4 showed membranous positivity, whereas DOG1 was negative in tumor cells at ×100 magnification. These findings provided ancillary support for secretory differentiation and helped exclude acinic cell carcinoma. AB-PAS staining showed positive intracytoplasmic secretory material in tumor cells at ×200 magnification. ETV6-NTRK3 fusion was confirmed by FISH in both tumors (75% and 80% of tumor nuclei), supporting the diagnosis of SCB. Both patients underwent breast-conserving surgery. Case 1 had no nodal metastasis (0/2; T1cN0M0, AJCC 8th edition), whereas Case 2 had sentinel lymph node metastasis (1/3; T2N1aM0, AJCC 8th edition), followed by axillary lymph node dissection (0/23). Both patients received adjuvant chemotherapy and radiation therapy. No locoregional recurrence or distant metastasis was observed during short-to-intermediate follow-up of 18 months in Case 1 and 30 months in Case 2. However, these observations reflect only the current clinical status of the two patients and are insufficient to support conclusions regarding long-term prognosis.

**Conclusions:**

These cases highlight the diagnostic challenges of SCB and underscore the importance of integrating morphology, immunohistochemistry, and FISH-based molecular confirmation. ETV6-NTRK3 fusion may represent a potential therapeutic option only in selected patients with advanced, unresectable, recurrent, or metastatic NTRK fusion-positive disease. Neither patient in the present report had advanced disease, received TRK inhibitor therapy, or underwent functional validation of the fusion. Therefore, no conclusion regarding therapeutic efficacy can be drawn from these two localized cases. Long-term follow-up remains necessary because late recurrence and distant metastasis have been reported in the literature, and the short-to-intermediate follow-up in the present cases does not allow definitive prognostic conclusions.

## 1 Introduction

Secretory carcinoma of the breast (SCB) is a rare special-type breast carcinoma recognized in the fifth edition of the World Health Organization Classification of Breast Tumours (1). McDivitt and Stewart first described this tumor in 1966 as juvenile breast carcinoma (4). SCB occurs across all age groups and demonstrates distinctive morphological features, including intracellular and extracellular secretory material (2, 4).

Immunohistochemically, SCB often shows a triple-negative phenotype with diffuse S-100 positivity (2). The hallmark molecular alteration is ETV6-NTRK3 gene fusion resulting from t(12;15)(p13;q25), which is highly useful for diagnosis in the appropriate breast tumor context (3, 5). In advanced or metastatic NTRK fusion-positive solid tumors, TRK inhibitors such as larotrectinib and entrectinib may represent therapeutic options (6, 7). This therapeutic statement should be interpreted cautiously in the present report because neither patient had advanced disease or received TRK inhibitor therapy.

Published Chinese literature on SCB includes a recent 52-patient prognostic study (8), a clinicopathological and immunophenotypic series of 15 cases (9), and a 44-patient study of pure secretory breast carcinoma (10). Individual Chinese case reports have also described uncommon clinical courses, including multiple distant brain metastases and pleural/mediastinal lymph node metastasis after long-term follow-up (11, 12). In this literature context, the present report adds two FISH-confirmed cases with detailed clinicopathological and molecular-pathological correlation. One case showed sentinel lymph node metastasis and lymphovascular invasion, whereas the other showed perineural invasion. These findings are not prominent features of classic SCB and may help expand the recognized clinicopathological spectrum of this rare tumor. They also emphasize the diagnostic value of integrating morphology, immunohistochemistry, and ETV6-NTRK3 fusion testing.

## 2 Case reports

### 2.1 Pathological and FISH methodology

All surgical specimens were processed according to the standardized protocol for breast cancer resection specimens. Briefly, the specimens were fixed in 4.0% neutral buffered formalin, serially sectioned at 5-mm intervals, sampled from representative tumor areas, routinely dehydrated, embedded in paraffin, sectioned at 4 μm, and stained with hematoxylin and eosin. Immunohistochemistry was performed using the EnVision two-step method. The immunohistochemical panel included ER, PR, HER2, Ki-67, S-100, CK5/6, SOX10, GATA3, p120, E-cadherin, p63, AR, CEA, and p53, as appropriate for each case. Mammaglobin, MUC4, and DOG1 immunostaining was performed on tumor tissue, and staining was evaluated in tumor cells at ×100 magnification. HER2 immunohistochemistry was performed using clone 4B5 and interpreted according to the ASCO/CAP HER2 testing criteria; an IHC score of 0 was interpreted as HER2-negative. The Ki-67 proliferation index was calculated as the percentage of invasive tumor cells showing nuclear staining in representative tumor areas. Multiple high-power fields, including areas of relatively increased labeling when present, were reviewed and averaged. Sentinel lymph nodes were assessed by routine serial sectioning and hematoxylin and eosin staining. All hematoxylin and eosin-stained slides and immunohistochemical slides were independently reviewed by two senior pathologists in a double-blinded manner, and the final diagnosis was confirmed by consensus.

Histological grading was performed according to the Nottingham grading system, which evaluates tubule formation, nuclear pleomorphism, and mitotic count. The total score was calculated by summing the three component scores and was used to assign the final histological grade. Case 1 showed a Nottingham score of 3 + 2 + 1 = 6, corresponding to Grade II. Case 2 showed a Nottingham score of 3 + 3 + 1 = 7, also corresponding to Grade II. TNM staging was assigned according to the eighth edition of the American Joint Committee on Cancer (AJCC) Cancer Staging Manual (13).

Tumor-infiltrating lymphocytes (TILs) were assessed on hematoxylin and eosin-stained sections according to the general principles of the International TILs Working Group recommendations (14). The invasive tumor boundary was first identified microscopically, and the stromal compartment within the invasive tumor bed was defined as the target area for evaluation. At low magnification, stromal immune cells, including lymphocytes and plasma cells, were identified within the tumor stroma. The TILs percentage was calculated as the proportion of stromal area occupied by TILs relative to the total stromal area within the invasive tumor bed, expressed as: TILs% = (stromal area containing TILs/total stromal area) × 100%. Multiple high-power fields were then reviewed, and the mean TILs percentage across representative fields was recorded.

Fluorescence *in situ* hybridization (FISH) for ETV6-NTRK3 fusion was performed using an ETV6-NTRK3 fusion detection kit purchased from Wuhan HealthCare Biotechnology Co., Ltd. The test was performed in the Department of Pathology, Zibo Central Hospital, a qualified hospital-based diagnostic pathology laboratory, according to the manufacturer’s instructions and institutional quality-control procedures. Fluorescence signals were interpreted according to the manufacturer’s instructions. Cells showing two red and two green signals were interpreted as negative. Cells showing one red signal, one green signal, and two fusion signals were interpreted as positive. ETV6-NTRK3 fusion was detected in 75% of tumor nuclei in Case 1 and 80% of tumor nuclei in Case 2. Therefore, the two tumors were regarded as SCB with molecular confirmation by FISH.

### 2.2 Case 1

A 55-year-old Chinese woman (Pathology No. 2024-28301) presented with a palpable mass in the left breast discovered during routine self-examination. Physical examination revealed a firm, mobile nodule approximately 1.5 cm in diameter in the upper outer quadrant of the left breast. Breast ultrasonography demonstrated a hypoechoic mass with irregular margins measuring 1.2 × 0.8 × 0.8 cm, classified as BI-RADS category IV.

Core needle biopsy revealed invasive carcinoma with abundant granular eosinophilic cytoplasm and intracellular vacuolization, consistent with secretory carcinoma. The patient subsequently underwent breast-conserving surgery (wide local excision) with sentinel lymph node biopsy.

**Figure 1:**
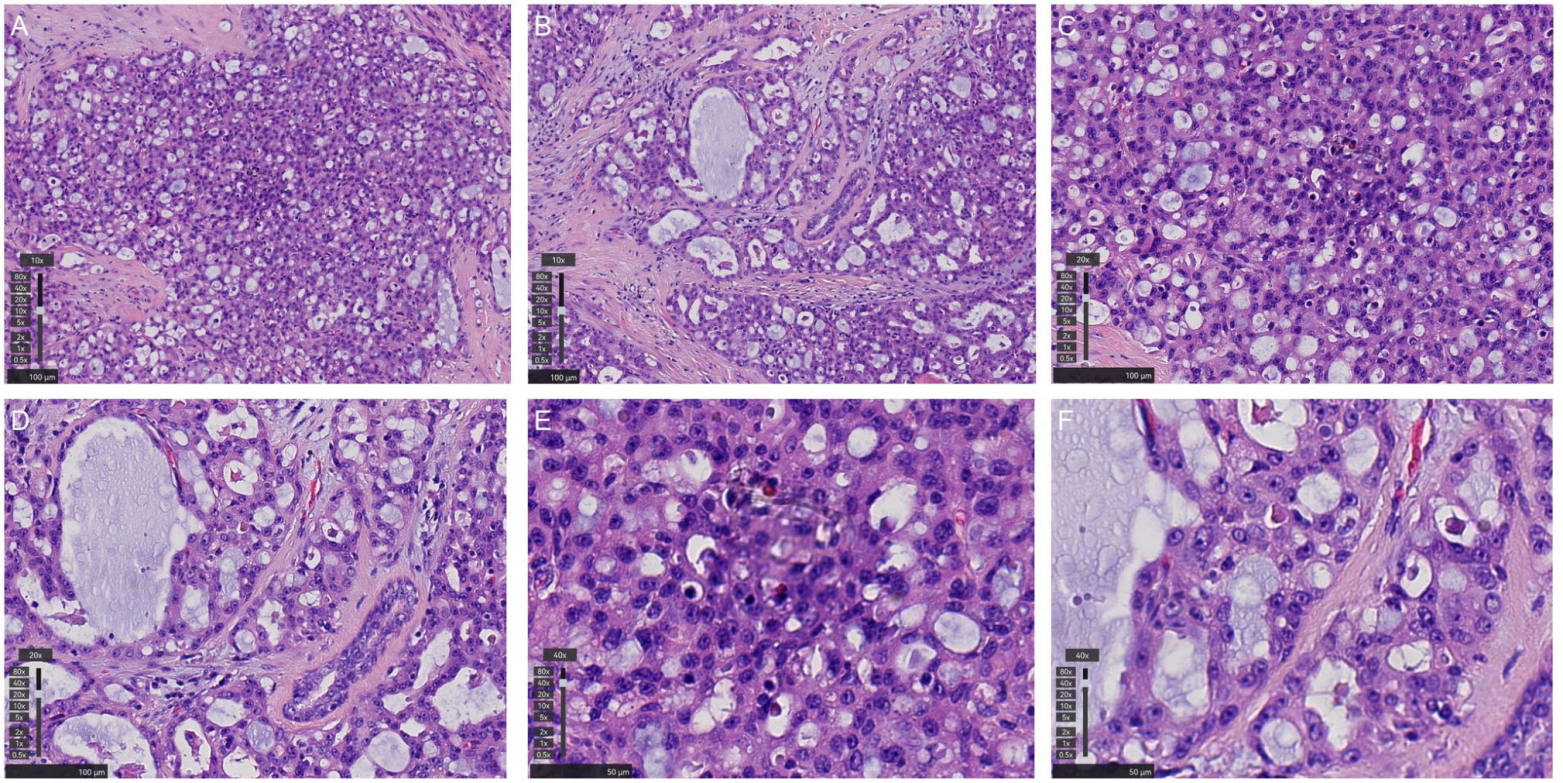
Histological features of Case 1. Hematoxylin and eosin (H&E) staining. Original magnifications: ×100 **(A, B)**, ×200 **(C, D)**, and ×400 **(E, F)**. Panels **(A, C, E)** show solid growth areas in the central region. Panels **(B, D, F)** show tubulocystic and papillary areas with abundant secretory material. Scale bars: 50 μm **(A, B)**, 25 μm **(C, D)**, and 10 μm **(E, F)**.

Gross examination revealed a well-circumscribed, firm, gray-white tumor measuring 1.2 × 0.8 × 0.8 cm. Microscopically, the tumor displayed tubulocystic and solid growth patterns with abundant intracellular and extracellular eosinophilic secretory material (**Figure 1**). The tumor was diagnosed as invasive secretory carcinoma, Nottingham Grade II, with interstitial lymphocytic infiltration (TILs: 1%). Perineural invasion was identified. The nipple and underlying tissue showed no significant involvement. Peripheral and deep resection margins were negative for tumor cells. Sentinel lymph node examination revealed 0/2 nodes positive for metastasis. The histological grade was further clarified using the Nottingham grading system: tubule formation score 3, nuclear pleomorphism score 2, and mitotic count score 1, giving a total score of 6 and corresponding to Grade II.

**Figure 2:**
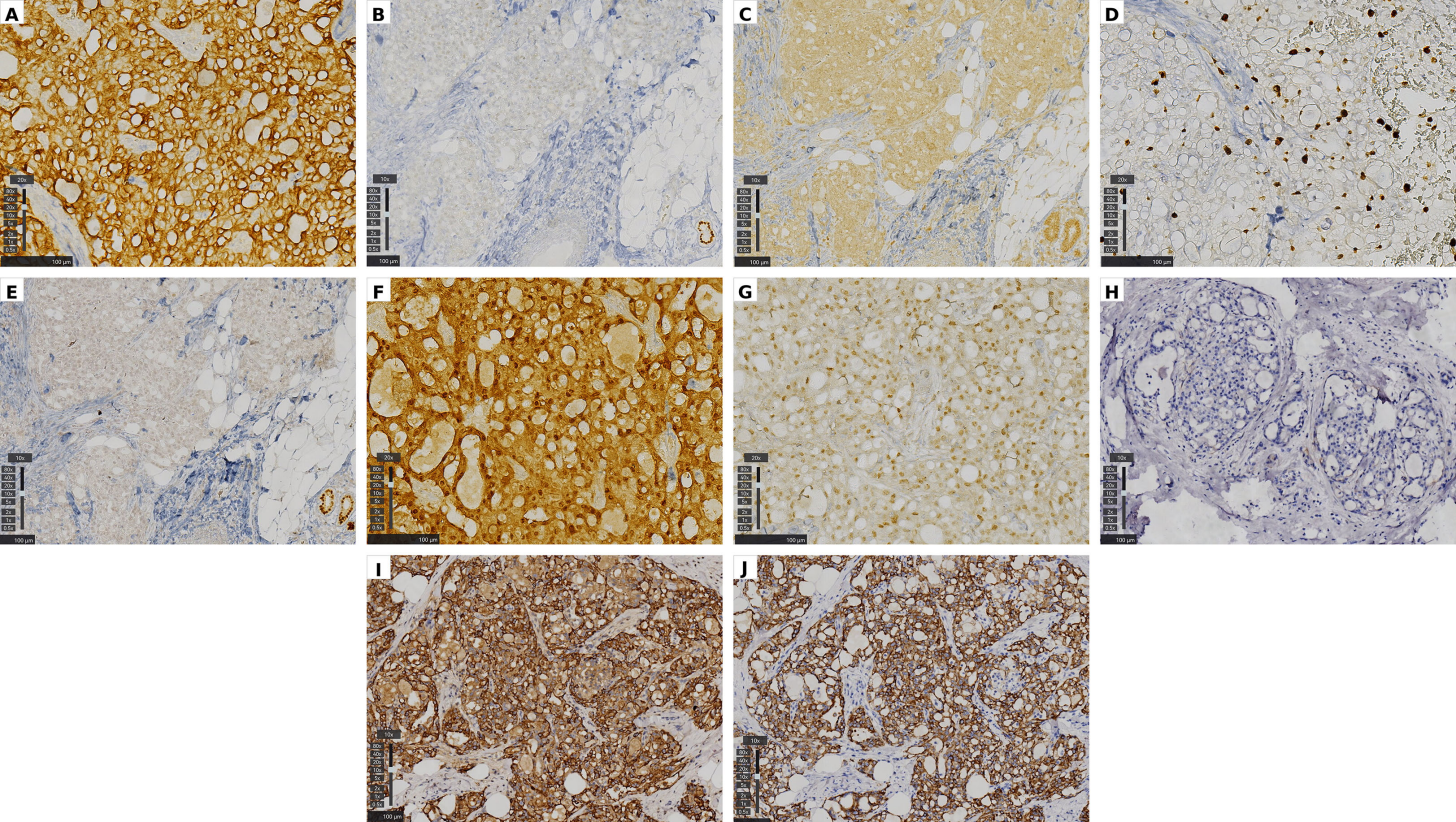
Immunohistochemical profile of Case 1. Immunohistochemical staining with DAB chromogen and hematoxylin counterstaining. Magnifications: 200× **(A, D, F, G)** and 100× **(B, C, E, H–J)**. Scale bars: 100 μm. **(A)** CK5/6 (clone D5/1684, Qiming Gene Technology Co., Ltd.) at 200× showing diffuse positivity in tumor cells. **(B)** ER (clone SP1, Roche Diagnostics) at 100× showing negative nuclear staining in tumor cells, with internal positive control present. **(C)** HER2 (clone 4B5, Roche Diagnostics) at 100× showing negative membranous staining in tumor cells, with internal positive control present. **(D)** Ki-67 (clone 30-9, Roche Diagnostics) at 200× showing nuclear positivity in tumor cells. **(E)** PR (clone 1E2, Roche Diagnostics) at 100× showing negative nuclear staining in tumor cells, with internal positive control present. **(F)** S-100 (clone Z0311, Qiming Gene Technology Co., Ltd.) at 200× showing diffuse positivity in tumor cells. **(G)** SOX10 (clone EP268, Fuzhou Maxin Biotechnology) at 200× showing diffuse nuclear positivity in tumor cells. **(H)** DOG1 (clone R072, Guangzhou Anbiping Medical Technology Co., Ltd.) at 100× showing negative staining in tumor cells. **(I)** Mammaglobin (clone R189, Guangzhou Anbiping Medical Technology Co., Ltd.) at 100× showing membranous positivity in tumor cells. **(J)** MUC4 (clone 8G7, Fuzhou Maixin Biotechnology Development Co., Ltd.) at 100× showing membranous positivity in tumor cells.

**Figure 3:**
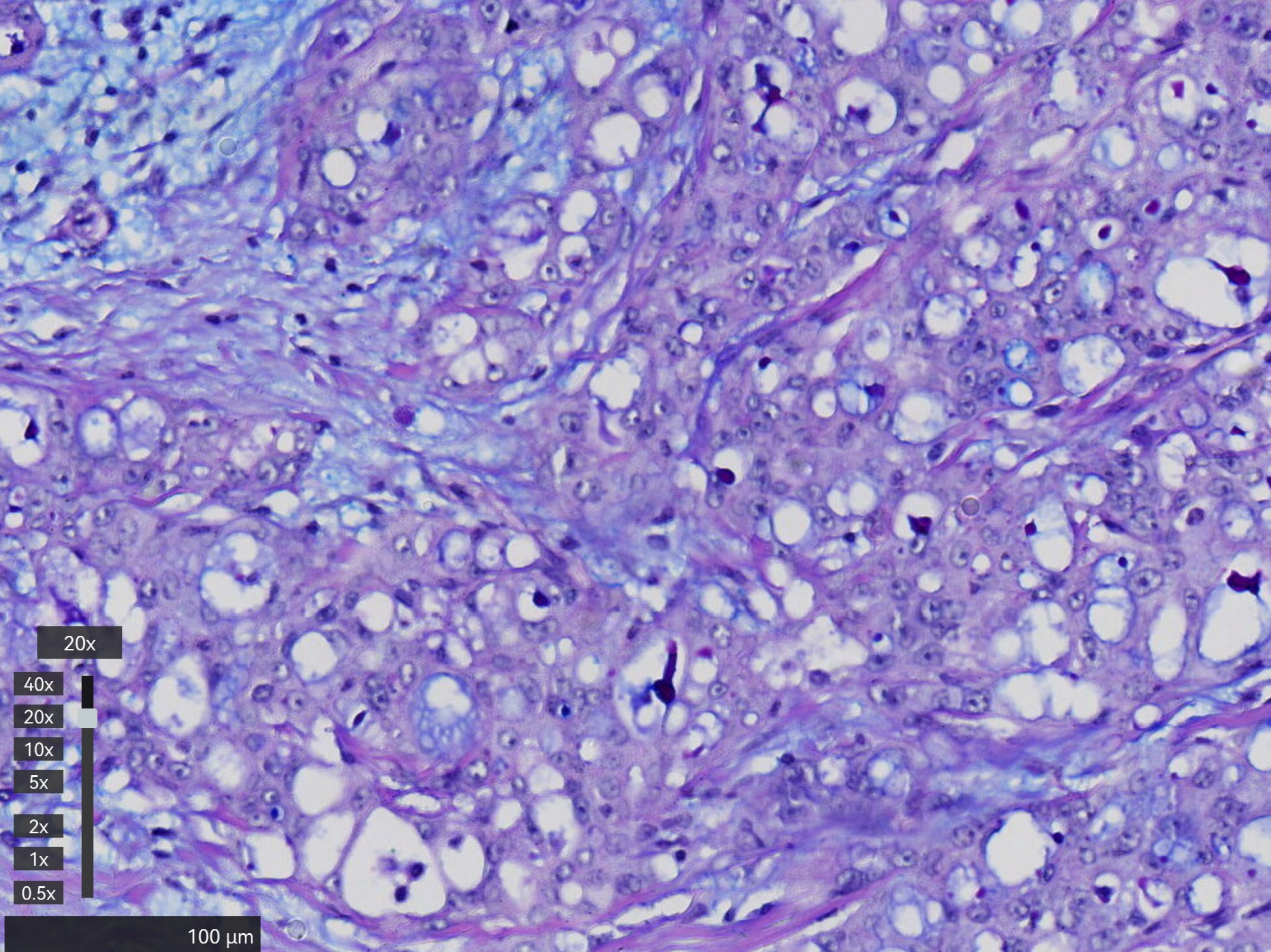
AB-PAS special staining of the tumor. AB-PAS special staining (Roche Diagnostics) at 200× magnification demonstrates abundant positive intracytoplasmic secretory material in tumor cells, supporting the presence of secretory differentiation. Scale bar: 100 μm.

**Figure 4:**
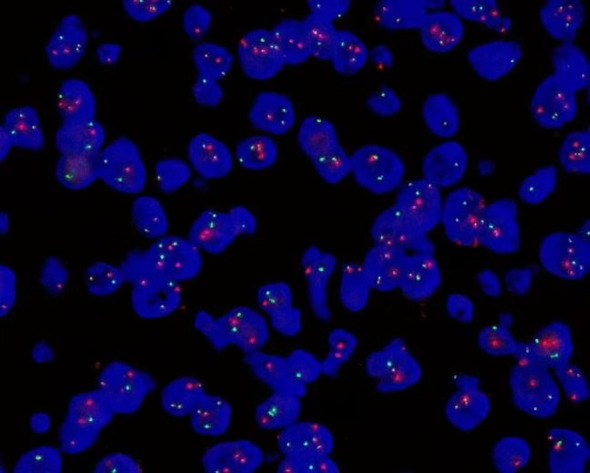
ETV6-NTRK3 gene fusion detection by fluorescence in situ hybridization (FISH) in Case 1. FISH was performed using an ETV6-NTRK3 fusion detection kit from Wuhan Kanglu Biotechnology Co., Ltd. (catalog no. CL-003; filing no. E-Han-Xie-Bei 20160084). Positive fusion signal patterns were observed in 75% of tumor cell nuclei, indicating the presence of ETV6-NTRK3 gene fusion. Interpretation criteria: negative = 2 red + 2 green signals; positive = 1 red + 1 green + 2 fusion signals.

Immunohistochemistry demonstrated: E-cadherin (+), GATA3 (-), p120 (+), S-100 (+), SOX10 (weak +), CK5/6 (+), p63 (-), ER (-), PR (-), AR (-), HER2 (4B5) (0), Ki-67 (+, 10%), mammaglobin (membranous +, ×100), MUC4 (membranous +, ×100), and DOG1 (-, ×100) (**Figure 2**). AB-PAS staining showed positive intracytoplasmic secretory material in tumor cells at ×200 magnification, further supporting secretory differentiation (**Figure 3**). Fluorescence *in situ* hybridization (FISH) confirmed ETV6-NTRK3 gene fusion in 75% of tumor nuclei (**Figure 4**).

The patient received adjuvant chemotherapy with the TC regimen (albumin-bound paclitaxel 260 mg/m² + cyclophosphamide 600 mg/m², 4 cycles), followed by radiation therapy (50 Gy in 25 fractions). At the most recent short-to-intermediate follow-up of 18 months, no locoregional recurrence or distant metastasis was observed. Continued long-term surveillance remains warranted.

### 2.3 Case 2

A 43-year-old Chinese woman (Pathology No. 2023-50580) presented with a palpable mass in the left breast. Physical examination revealed a firm mass approximately 4 cm in diameter in the upper outer quadrant of the left breast.

Ultrasonography showed a hypoechoic mass measuring 3.8 × 3.0 × 2.2 cm, BI-RADS IV. Core needle biopsy confirmed invasive secretory carcinoma with prominent stromal lymphocytic infiltration.

The patient underwent breast-conserving surgery with sentinel lymph node biopsy and axillary dissection. Gross examination revealed a solid-cystic, gray-white tumor measuring 3.8 × 3.0 × 2.2 cm. Microscopy demonstrated solid, tubulocystic, and papillary growth patterns with abundant intracellular and extracellular eosinophilic secretory material. The tumor was diagnosed as invasive secretory carcinoma with basal-like breast carcinoma features, focal necrosis, and prominent stromal lymphocytic and plasmacytic infiltration. Lymphovascular invasion was identified. The tumor invaded the dermis but not the epidermis or perineural structures. Sentinel node metastasis was identified (1/3), whereas 0/23 axillary nodes were involved. All resection margins (superior, inferior, medial, lateral, superficial, and deep) were negative for tumor cells. Histological grading according to the Nottingham system showed a tubule formation score of 3, nuclear pleomorphism score of 3, and mitotic count score of 1, giving a total score of 7 and corresponding to Grade II.

Immunohistochemistry showed: S-100 (+), CK5/6 (+), p63 (-), CEA (-), ER (-), PR (-), AR (-), HER2 (4B5) (0), p53 (focal +, 10%), and Ki-67 (+, 12%). Mammaglobin and MUC4 showed tumor-cell membranous positivity, whereas DOG1 was negative at ×100 magnification. FISH analysis demonstrated ETV6-NTRK3 fusion in 80% of tumor nuclei.

The patient received adjuvant chemotherapy with AC followed by T regimen (doxorubicin 60 mg/m² + cyclophosphamide 600 mg/m², 4 cycles, followed by albumin-bound paclitaxel 260 mg/m², 4 cycles) plus radiation therapy (50 Gy in 25 fractions). At the most recent short-to-intermediate follow-up of 30 months, no locoregional recurrence or distant metastasis was observed. Continued long-term surveillance remains warranted.

## 3 Discussion

### 3.1 Overview of SCB

Secretory carcinoma of the breast (SCB) is a rare special-type breast carcinoma characterized by distinctive secretory morphology and recurrent ETV6-NTRK3 fusion (1–3). Published clinicopathological series and population-based studies generally suggest relatively indolent behavior and favorable survival compared with conventional high-grade triple-negative breast carcinoma, but nodal involvement, local recurrence, and distant metastasis have been reported (8–12, 15–17). Prognosis should therefore be interpreted cautiously, particularly in tumors with larger size, lymphovascular invasion, perineural invasion, nodal metastasis, or limited follow-up. The present report adds two FISH-confirmed Chinese cases: one with perineural invasion and one with lymphovascular invasion and sentinel lymph node metastasis, supporting the value of integrated clinicopathological and molecular assessment.

### 3.2 Clinical features

SCB predominantly affects females, although it can occur across a wide age range from children to elderly patients (4, 18). The typical clinical presentation is a slow-growing, well-circumscribed, mobile, painless breast mass, most commonly located in the periareolar region or upper outer quadrant (2, 18). Axillary lymph node metastasis is uncommon but has been documented in both case series and individual reports (8–10, 15). Our two cases were consistent with these clinical features: both patients presented with palpable masses in the upper outer quadrant of the left breast, and Case 2 had limited nodal involvement involving one sentinel lymph node (**Table 1**).

**Table 1:** .

Characteristics of two cases	Case 1	Case 2
Age (years)	55	43
Sex	Female	Female
Pathology No.	2024-28301	2023-50580
TNM stage (AJCC 8th edition)	T1cN0M0	T2N1aM0
Clinical presentation	Palpable left breast mass	Palpable left breast mass
Tumor size (cm)	1.2 × 0.8 × 0.8	3.8 × 3.0 × 2.2
Location	Left breast, upper outer quadrant	Left breast, upper outer quadrant
Histological diagnosis	Secretory carcinoma, Grade II	Invasive secretory carcinoma with basal-like features
Nottingham score	3 + 2 + 1 = 6, Grade II	3 + 3 + 1 = 7, Grade II
TILs (%)	1	2
Lymphovascular invasion	Absent	Present
Perineural invasion	Present	Absent
Sentinel lymph nodes	0/2	1/3
Axillary lymph nodes	N/A	0/23
ER	Negative	Negative
PR	Negative	Negative
HER2	Negative (0)	Negative (0)
S-100	Positive	Positive
Mammaglobin	Positive (membranous)	Positive (membranous)
MUC4	Positive (membranous)	Positive (membranous)
DOG1	Negative	Negative
CK5/6	Positive	Positive
Ki-67 (%)	10	12
ETV6-NTRK3 FISH	Positive (75%)	Positive (80%)
Chemotherapy	TC (Albumin-bound paclitaxel + cyclophosphamide)	AC-T (doxorubicin + cyclophosphamide → Albumin-bound paclitaxel)
Radiation therapy	50 Gy/25 fractions	50 Gy/25 fractions
Follow-up	18 months, no locoregional recurrence or distant metastasis observed during short-to-intermediate follow-up	30 months, no locoregional recurrence or distant metastasis observed during short-to-intermediate follow-up

### 3.3 Pathological features

SCB demonstrates characteristic histological patterns, including: (1) microcystic/tubulocystic growth, with tumor cells arranged in cyst-like spaces containing eosinophilic secretions; (2) papillary growth, with papillary structures containing fibrovascular cores; and (3) solid growth, with solid nests or sheets of tumor cells (2, 19). Hallmark features include abundant granular eosinophilic or vacuolated cytoplasm and intracellular/extracellular eosinophilic secretory material on hematoxylin and eosin staining (2, 19). AB-PAS staining showed positive intracytoplasmic secretory material in tumor cells at ×200 magnification. This result is consistent with the classical histochemical features of SCB, in which intracellular and/or extracellular secretory material may be highlighted by PAS, PAS-D, Alcian blue, or combined Alcian blue/PAS staining. Together with the characteristic secretory morphology on hematoxylin and eosin staining, triple-negative immunophenotype, S-100 and CK5/6 positivity, and FISH-confirmed ETV6-NTRK3 fusion, the positive AB-PAS result further supports the diagnosis of SCB.

According to previous morphological descriptions, SCB tumor cells may show eosinophilic granular cytoplasm or prominent cytoplasmic vacuolization (2, 19). In Case 1, tumor cells showed predominantly eosinophilic granular cytoplasm, whereas Case 2 showed both eosinophilic and vacuolated cells. The tumor cells generally showed minimal cytologic atypia and rare mitotic figures, consistent with the low-grade morphology commonly observed in SCB (2, 19). Nottingham grading was also applied to both tumors to provide a standardized histological assessment. Both cases were classified as Grade II, with scores of 6 and 7, respectively, reflecting limited tubule formation, variable nuclear pleomorphism, and low mitotic activity.

Stromal features of SCB may include fibrosis, myxoid change, chronic inflammatory cell infiltration, and, rarely, hemorrhage or necrosis (2). In the present cases, perineural invasion was identified in Case 1, whereas lymphovascular invasion and sentinel lymph node metastasis were identified in Case 2. Perineural invasion and lymphovascular invasion are not dominant features of classic SCB, which is generally regarded as an indolent special-type breast carcinoma. Nevertheless, these findings may have clinical and prognostic relevance. Perineural invasion indicates the ability of tumor cells to spread along nerve sheaths. In breast cancer, it has been associated with more aggressive biological behavior, possible higher-grade transformation, local extension, and unfavorable prognosis. Its presence may therefore influence local treatment considerations, including careful assessment of surgical margins and the need for postoperative radiotherapy in selected patients. Lymphovascular invasion is associated with an increased risk of lymph node metastasis and is generally regarded as an adverse prognostic factor. This is consistent with the sentinel lymph node metastasis observed in Case 2. The independent prognostic value of perineural invasion and lymphovascular invasion in SCB remains uncertain because of the rarity of this tumor. Even so, their presence suggests a relatively more aggressive phenotype and supports closer surveillance and individualized consideration of adjuvant therapy. Published series and case reports have documented nodal involvement and rare distant metastases in a subset of patients with SCB. These data support careful pathological assessment and long-term follow-up, particularly in cases with lymphovascular invasion, perineural invasion, larger tumor size, or nodal metastasis (8–12, 15).

### 3.4 Immunohistochemical profile

SCB exhibits a distinctive immunohistochemical profile that differs from conventional invasive ductal carcinoma (2, 16). The typical immunophenotype includes:

Triple-negative phenotype: SCB is frequently negative for estrogen receptor (ER), progesterone receptor (PR), and HER2, although immunophenotypic variation may occur (2, 16). Both of our cases showed an ER-negative, PR-negative, and HER2-negative phenotype.

S-100 protein: Diffuse positivity for S-100 is a characteristic feature of SCB and is useful in distinguishing it from many conventional triple-negative breast cancers (2, 16). Both our cases demonstrated S-100 positivity.

Basal markers: CK5/6 is often positive, supporting basal-like differentiation (2, 16). Both our cases showed CK5/6 positivity.

Ki-67 proliferation index is generally low in SCB, indicating slow-growing tumors (2). However, our cases showed slightly higher Ki-67 indices (10% and 12%), which may warrant close follow-up.

Pan-TRK immunohistochemistry has been reported as a useful screening marker for NTRK fusion-associated tumors, including SCB, and may provide a rapid adjunct to molecular testing (20). Ancillary markers, including mammaglobin, MUC4, and GCDFP-15, may help support secretory differentiation. DOG1 and NR4A3 may assist in the differential diagnosis of acinic cell carcinoma and other histological mimics in selected settings. In both cases, mammaglobin and MUC4 showed tumor-cell membranous positivity at ×100 magnification, supporting secretory differentiation. DOG1 was negative at ×100 magnification, arguing against acinic cell carcinoma. Pan-TRK, GCDFP-15, and NR4A3 immunostaining were not performed; therefore, we did not rely on Pan-TRK immunohistochemistry or a fully comprehensive contemporary immunohistochemical panel for diagnostic interpretation. The diagnosis was based on integrated findings, including characteristic morphology, triple-negative phenotype, S-100 and CK5/6 positivity, AB-PAS-positive intracytoplasmic secretory material, mammaglobin and MUC4 positivity, DOG1 negativity, absence of myoepithelial marker expression in invasive areas, and molecular confirmation of ETV6-NTRK3 fusion by FISH.

### 3.5 Molecular confirmation and literature-based context

The defining molecular alteration in SCB is ETV6-NTRK3 fusion resulting from t(12;15)(p13;q25) (3, 5). In our cases, FISH detected the fusion in 75% and 80% of tumor nuclei. In a primary breast tumor with classic secretory morphology and a supportive immunophenotype, this finding provides strong molecular confirmation of SCB.

FISH has important limitations. It confirms the fusion signal but does not define the transcript, breakpoint, transcript abundance, genomic landscape, pathway activation, or functional consequences. Therefore, the present report is limited to molecular confirmation by FISH. RNA sequencing, next-generation sequencing, pathway-specific assays, and functional validation were not performed.

Published molecular and clinical studies are summarized in **Table 2**. We use these studies only as background for ETV6-NTRK3-positive SCB, pathway biology, transcriptomic testing, and TRK inhibitor therapy. These data were not generated from the present cases.

**Table 2:** Simplified summary of selected SCB studies and their relevance to the present cases.

Study/evidence	Evidence type	Main message	Use in present report
Lae et al. (19)	SCB series	Classic morphology, basal-like immunophenotype, and ETV6-NTRK3 fusion.	Supports integrated diagnosis by morphology, IHC, and FISH.
Hoda et al. (15); Jacob et al. (16); Sanli et al. (17)	Clinicopathological and population-based studies	SCB is often indolent, but nodal or distant metastasis can occur.	Supports cautious prognostic interpretation.
Li et al. (10); Zhao et al. (8)	Chinese cohorts	Provide regional clinicopathological and prognostic data.	Places the two Chinese cases in context.
Krings et al. (5)	Genomic profiling	Reported a relatively simple genomic landscape in many SCBs.	Provides molecular context only; genomic profiling was not performed here.
Maund et al. (21); Kinnunen et al. (22); Guha et al. (23)	NTRK fusion and transcriptomic studies	Show that RNA-based or broader molecular analyses may provide information beyond FISH.	Highlights the limitation of FISH-only confirmation.
Present report	Two FISH-confirmed cases	Morphology, IHC, AB-PAS staining, DOG1 negativity, and FISH supported SCB; PNI/LVI/nodal involvement were observed.	Adds clinicopathological correlation without comprehensive molecular profiling or functional validation.

### 3.6 Differential diagnosis

SCB can overlap morphologically with several benign and malignant breast lesions, including secretory, microcystic, tubular, apocrine, basaloid, and mucin-rich tumors (2, 19). In our cases, the diagnosis was supported by classic secretory morphology, a triple-negative phenotype, S-100 and CK5/6 positivity, absence of myoepithelial marker expression in invasive areas, mammaglobin and MUC4 membranous positivity, DOG1 negativity, and molecular confirmation of ETV6-NTRK3 fusion by FISH.

Acinic cell carcinoma is an important differential diagnosis. It may show microglandular or solid architecture, granular cytoplasm, and a triple-negative phenotype. However, it usually shows serous acinar differentiation and may express DOG1, lysozyme, amylase, or other acinar markers; ETV6-NTRK3 fusion is not characteristic (24). In our cases, neither tumor showed convincing serous acinar differentiation. Both tumors were DOG1-negative at ×100 magnification and showed classic SCB morphology, S-100/CK5/6 positivity, mammaglobin/MUC4 membranous positivity, AB-PAS-positive intracytoplasmic secretory material, and FISH-confirmed ETV6-NTRK3 fusion. These findings favored SCB over acinic cell carcinoma.

Microglandular adenosis-associated carcinoma may show infiltrative small glands and S-100 positivity. In contrast to SCB, it typically consists of open tubules lined by a single epithelial layer with basement membrane material (25). The solid, tubulocystic, and papillary secretory architecture in our cases, together with FISH-based molecular confirmation, favored SCB.

Apocrine carcinoma may mimic SCB because of abundant eosinophilic granular cytoplasm. However, apocrine carcinoma usually shows apocrine cytologic features, androgen receptor expression, and possible GCDFP-15 expression; it lacks the characteristic ETV6-NTRK3 fusion of SCB (26). In our cases, both tumors were AR-negative, showed typical secretory architecture, and demonstrated ETV6-NTRK3 fusion by FISH.

Adenoid cystic carcinoma is another relevant mimic because it may be triple-negative and basaloid. It usually shows cribriform or tubular architecture with true glandular and pseudoluminal spaces containing basement membrane-like material. It is also commonly associated with MYB or MYBL1 alterations rather than ETV6-NTRK3 fusion (27). In our cases, the absence of a classic adenoid cystic pattern and the presence of ETV6-NTRK3 fusion supported SCB.

Other differential diagnoses include lactating adenoma, juvenile papillomatosis, mucinous carcinoma, solid papillary carcinoma, and conventional triple-negative invasive carcinoma. These entities can usually be separated from SCB by their architecture, myoepithelial pattern, receptor profile, mucin or neuroendocrine differentiation, and lack of ETV6-NTRK3 fusion. Although Pan-TRK, GCDFP-15, and NR4A3 were not performed, the integrated morphology, available immunophenotype, DOG1 negativity, and FISH findings supported SCB.

Overall, these findings support an integrated diagnostic approach rather than reliance on morphology alone, particularly when secretory, apocrine, acinic-like, or basaloid features overlap (2, 19, 24–27).

### 3.7 Treatment and prognosis

Surgical excision with negative margins remains the mainstay of treatment for localized SCB. Because disease-specific prospective trials are lacking, adjuvant treatment is individualized according to conventional breast cancer risk factors, including tumor size, nodal status, histological grade, lymphovascular invasion, and receptor status (8–10, 16, 17). In our patients, adjuvant radiotherapy followed breast-conserving surgery. Chemotherapy was selected after multidisciplinary discussion because both tumors were triple-negative and Grade II, and Case 2 had additional risk factors, including larger tumor size, sentinel lymph node metastasis, and lymphovascular invasion.

The chemotherapy regimens were chosen according to institutional breast cancer practice and the 2024 CSCO Breast Cancer Guidelines (28). Case 2 received an anthracycline- and taxane-based regimen because of higher pathological risk. Platinum-based or dose-dense chemotherapy was not used because the tumors were localized and SCB is biologically distinct from conventional high-grade triple-negative breast cancer. In addition, platinum-containing adjuvant chemotherapy was not a standard postoperative option for such patients in the referenced CSCO guidance (28).

No locoregional recurrence or distant metastasis was observed after 18 months in Case 1 or 30 months in Case 2. These findings describe only the current disease status and do not establish a favorable long-term prognosis. Because nodal involvement, local recurrence, and rare late metastasis have been reported (11, 12, 15), continued surveillance is warranted, particularly in patients with adverse pathological features.

### 3.8 Limitations

Several limitations should be acknowledged. This report includes only two cases with short-to-intermediate follow-up, so no definitive conclusions regarding prognosis, recurrence risk, or optimal treatment can be drawn. The immunohistochemical panel was not fully comprehensive because Pan-TRK, GCDFP-15, and NR4A3 were not performed, although mammaglobin, MUC4, DOG1, AB-PAS staining, and FISH supported the final diagnosis. Additional molecular analyses, including RNA sequencing, next-generation sequencing, transcriptomic profiling, pathway assays, and functional validation, were also unavailable. Therefore, all pathway-related and therapeutic discussions should be interpreted strictly as literature-based background rather than evidence generated by the present cases.

### 3.9 Conclusions

We report two FISH-confirmed cases of SCB with classic morphology, supportive immunophenotype, AB-PAS-positive secretory material, mammaglobin/MUC4 positivity, DOG1 negativity, and ETV6-NTRK3 fusion. Perineural invasion in one case and lymphovascular invasion with sentinel lymph node metastasis in the other broaden the clinicopathological context of this rare tumor. ETV6-NTRK3 fusion primarily served as diagnostic molecular confirmation in these localized cases; it did not justify TRK inhibitor therapy and does not provide evidence of treatment efficacy. Because follow-up remains limited and late recurrence or metastasis has been reported, larger studies with longer follow-up and broader molecular profiling are needed.

## Data Availability

The raw data supporting the conclusions of this article will be made available by the authors, without undue reservation.
